# Supplementary material for: Determinants of the COVID-19 vaccine hesitancy spectrum
Source: PLoS One. 2022 Jun 1;17(6):e0267734. doi: 10.1371/journal.pone.0267734 (PMC9159626; doi:10.1371/journal.pone.0267734)
Supplement: S1 Table — (DOCX) [file pone.0267734.s001.docx]

**Supplemental Material**

**Table 1S. Unadjusted multinomial regression of unvaccinated groups compared to fully/partially vaccinated group as a reference category**

|  |  | **Relative risk of each unvaccinated group compared to fully/partially vaccinated group [RRR, 95%CI]** | | | |  |
| --- | --- | --- | --- | --- | --- | --- |
|  |  | **Eager-to-take** | **Wait-and-see** | **Undecided** | **Refuse** |  |
| **Demographic characteristics** | |  |  |  |  |  |
| **Age groups** | |  |  |  |  |  |
| *18-29* | | 12.75 [7.39, 22.00] * | 11.08 [6.38, 19.24] * | 6.02 [3.64, 9.97] * | 3.80 [2.44, 5.92] * |  |
| *30-49* | | 8.50 [5.07, 14.26] * | 7.91 [4.71, 13.29] * | 6.11 [3.90, 9.56] * | 4.25 [2.93, 6.18] * |  |
|  | *50-64* | 4.04 [2.34, 6.98] * | 3.00 [1.71, 5.28] * | 3.56 [2.22, 5.70] * | 2.22 [1.48, 3.33] * |  |
|  | *65+* | - | - | - | - |  |
| **Gender** |  |  |  |  |  |  |
|  | *Female* | - | - | - | - |  |
|  | *Male* | 0.82 [0.64, 1.04] | 1.18 [0.91, 1.52] | 1.41 [1.10, 1.80] * | 1.62 [1.28, 2.07] * |  |
| **Race/Ethnicity** | |  |  |  |  |  |
|  | *White Non-Hispanic* | - | - | - | - |  |
|  | *Black Non-Hispanic* | 0.90 [0.62, 1.29] | 2.02 [1.48, 2.77] * | 1.40 [1.03, 1.91] * | 1.29 [0.95, 1.73] |  |
|  | *Hispanic* | 2.02 [1.49, 2.75] * | 2.11 [1.51, 2.93] * | 1.66 [1.21, 2.28] * | 1.34 [0.97, 1.83] |  |
|  | *Other* | 1.93 [1.30, 2.86] * | 0.68 [0.36, 1.30] | 0.37 [0.18, 0.78] * | 0.32 [0.15, 0.66] * |  |
| **Education** |  |  |  |  |  |  |
| *Less than/graduated high school* | | 2.75 [1.96, 3.87] * | 3.29 [2.19, 4.93] * | 6.56 [3.49, 9.79] * | 5.89 [4.06, 8.53] * |  |
| *Some college or graduated college* | | 1.41 [1.05, 1.89] * | 2.53 [1.80, 3.55] * | 3.24 [2.25, 4.68] * | 2.75 [1.96, 3.85] * |  |
| *Post-graduate/professional* | | - | - | - | - |  |
| **Employment status** | |  |  |  |  |  |
|  | *Unemployed* | 0.99 [0.77, 1.27] | 0.76 [0.57, 0.99] * | 0.72 [0.55, 0.94] * | 0.86 [0.67, 1.10] |  |
| *Employed (full or part time)* | | - | - | - | - |  |
| **Annual Income** | |  |  |  |  |  |
| *Less than $25,000* | | 2.40 [1.67, 3.44] * | 3.58 [2.42, 5.29] * | 5.64 [3.71, 8.58] * | 3.93 [2.76, 5.61] * |  |
| *$25,000 to less than $50,000* | | 1.68 [1.19, 2.35] * | 2.13 [1.45, 3.12] * | 3.57 [2.38, 5.36] * | 1.91 [1.33, 2.73] * |  |
| *$50,000 to less than $75,000* | | 0.97 [0.66, 1.42] | 1.37 [0.90, 2.07] | 2.57 [1.68, 3.93] * | 1.36 [0.93, 1.99] |  |
| *$75,000 to less than $100,000* | | 0.94 [0.62, 1.42] | 1.31 [0.84, 2.05] | 2.50 [1.60, 3.90] * | 1.40 [0.94, 2.09] |  |
|  | *$100,000 or more* | - | - | - | - |  |
| **Religion** |  |  |  |  |  |  |
|  | *Protestant* | - | - | - | - |  |
|  | *Evangelical* | 1.17 [0.60, 2.26] | 1.59 [0.97, 2.61] | 1.56 [0.96, 2.54] | 1.42 [0.89, 2.26] |  |
| *Catholic, Roman Catholic* | | 1.52 [1.02, 2.26] * | 0.53 [0.35, 0.80] * | 0.79 [0.55, 1.13] | 0.56 [0.39, 0.80] * |  |
|  | *Other* | 1.07 [0.70, 1.63] | 0.68 [0.46, 1.00] | 0.73 [0.50, 1.05] | 0.75 [0.54, 1.05] |  |
| *Nothing in particular/Atheist/Agnostic* | | 2.04 [1.42, 2.94] * | 0.81 [0.57, 1.15] | 0.79 [0.56, 1.11] | 0.66 [0.47, 0.91] * |  |
| **Metro Status** | |  |  |  |  |  |
| *Non-metro/rural* | | - | - | - | - |  |
|  | *Metro* | 1.89 [1.28, 2.79] * | 1.15 [0.81, 1.64] | 0.79 [0.59, 1.07] | 0.53 [0.41, 0.69] * |  |
| **Census region** | |  |  |  |  |  |
|  | *North East* | - | - | - | - |  |
|  | *North Central* | 0.71 [0.49, 1.02] | 1.18 [0.78, 1.79] | 1.28 [0.84, 1.94] | 1.68 [1.13, 2.51] * |  |
|  | *South* | 0.61 [0.44, 0.83] * | 1.36 [0.94, 1.96] | 1.55 [1.07, 2.24] * | 1.70 [1.18, 2.45] * |  |
|  | *West* | 0.76 [0.54, 1.08] | 0.83 [0.54, 1.29] | 1.41 [0.94, 2.12] | 1.32 [0.88, 1.98] |  |
| **Health insurance type** | |  |  |  |  |  |
|  | *Private* | - | - | - | - |  |
|  | *Medicare* | 0.25 [0.16, 0.38] * | 0.35 [0.23, 0.53] * | 0.46 [0.32, 0.66] * | 0.53 [0.37, 0.76] * |  |
|  | *Medicaid* | 2.10 [1.50, 2.95] * | 2.36 [1.66, 3.35] * | 2.02 [1.42, 2.88] * | 3.16 [2.28, 4.38] * |  |
| *TRICARE/ Veterans/Indian HS/ Other* | | 1.08 [0.65, 1.80] | 1.17 [0.69, 1.99] | 1.24 [0.75, 2.04] | 1.76 [1.11, 2.78] |  |
|  | *Uninsured* | 4.37 [2.74, 6.98] * | 4.09 [2.47, 6.76] * | 4.76 [2.98, 7.61] * | 7.01 [4.50, 10.91] * |  |
| **Parent** | |  |  |  |  |  |
|  | *No* | - | - | - | - |  |
|  | *Yes* | 1.81 [1.40, 2.34] * | 2.27 [1.74, 2.97] * | 2.13 [1.64, 2.75] * | 3.11 [2.44, 3.96] * |  |
| **Political party** | |  |  |  |  |  |
|  | *Democrat* | - | - | - | - |  |
|  | *Republican* | 0.78 [0.54, 1.14] | 2.46 [1.75, 3.46] * | 3.39 [2.45, 4.67] * | 5.64 [4.04, 7.87] * |  |
|  | *Independent* | 1.69 [1.29, 2.21] * | 2.23 [1.62, 3.05] * | 2.36 [1.73, 3.23] * | 3.26 [2.33, 4.56] * |  |
|  | *Other, don’t know, refused* | 1.50 [0.83, 2.71] | 2.48 [1.35, 4.56] * | 3.19 [1.82, 5.59] * | 5.66 [3.34, 9.59] * |  |
| **COVID exposure/experience** | |  |  |  |  |  |
| **Had COVID-19** | |  |  |  |  |  |
|  | *No* | - | - | - | - |  |
|  | *Yes* | 1.44 [1.02, 2.02] * | 1.44 [1.00, 2.06] | 1.56 [1.12, 2.18] * | 1.97 [1.45, 2.67] * |  |
| **Personally know anyone died of COVID-19** | |  |  |  |  |  |
| *No* | | - | - | - | - |  |
|  | *Yes* | 0.70 [0.55, 0.90] * | 0.66 [0.51, 0.86] * | 0.72 [0.57, 0.93] * | 0.56 [0.43, 0.71] * |  |
| **Financial severity (mean, SD) **** | | 1.40 [1.20, 1.63] * | 1.72 [1.48, 1.99] * | 1.74 [1.51, 2.00] * | 1.64 [1.42, 1.88] * |  |

* p-value <0.05

** Financial severity is an index score of financial hardships including losing a job, losing income, trouble paying for necessities. The minimum is 0, indicating no financial hardship and maximum is 3 indicating hardships in all three areas.
