# Supplementary material for: Correction: Determinants of the COVID-19 vaccine hesitancy spectrum
Source: PLoS One. 2025 Feb 21;20(2):e0315702. doi: 10.1371/journal.pone.0315702 (PMC11844853; doi:10.1371/journal.pone.0315702)
Supplement: S1 File — (PDF) [file pone.0315702.s001.pdf]

PLOS ONE

RESEARCH ARTICLE

Determinants of the COVID-19 vaccine hesitancy spectrum

Rachael Piltch-Loeb[1]*, Diana R. Silver[2], Yeerae Kim[1], Hope Norris[2], Elizabeth McNeill[2], David M. Abramson[1]

1 Center for Public Health Disaster Science, School of Global Public Health, New York University, New York, New York, United States of America, **2** Department of Public Health Policy and Management, School of Global Public Health, New York University, New York, New York, United States of America

* rpl5@nyu.edu

Check for updates

OPEN ACCESS

Citation: Piltch-Loeb R, Silver DR, Kim Y, Norris H, McNeill E, Abramson DM (2022) Determinants of the COVID-19 vaccine hesitancy spectrum. PLoS ONE 17(6): e0267734. https://doi.org/10.1371/journal.pone.0267734

Editor: Janet E Rosenbaum, SUNY Downstate Health Sciences University, UNITED STATES

Received: August 10, 2021

Accepted: April 13, 2022

Published: June 1, 2022

Copyright: © 2022 Piltch-Loeb et al. This is an open access article distributed under the terms of the Creative Commons Attribution License, which permits unrestricted use, distribution, and reproduction in any medium, provided the original author and source are credited.

Data Availability Statement: ***PA @ ACCEPT *** Data will be uploaded to ICPSR COVID-19 data repository upon acceptance of the manuscript.

Funding: Funding for this research was provided by a grant from the National Science Foundation (Grant #2049886). The funders had no role in study design, data collection and analysis, decision to publish, or preparation of the manuscript.

Competing interests: The authors have declared that no competing interests exist.

Abstract

Vaccine hesitancy remains an issue in the United States. This study conducted an online survey [N = 3,013] using the Social Science Research Solution [SSRS] Opinion Panel web panelists, representative of U.S. adults age 18 and older who use the internet, with an over-sample of rural-dwelling and minority populations between April 8 and April 22, 2021- as vaccine eligibility opened to the country. We examined the relationship between COVID-19 exposure and socio-demographics with vaccine intentions [eager-to-take, wait-and-see, undecided, refuse] among the unvaccinated using multinomial logistic regressions [ref: fully/partially vaccinated]. Results showed vaccine intentions varied by demographic characteristics and COVID-19 experience during the period that eligibility for the vaccine was extended to all adults. At the time of the survey approximately 40% of respondents were unvaccinated; 41% knew someone who had died of COVID-19, and 38% had experienced financial hardship as a result of the pandemic. The vaccinated were more likely to be highly educated, older adults, consistent with the United States initial eligibility criteria. Political affiliation and financial hardship experienced during the pandemic were the two most salient factors associated with being undecided or unwilling to take the vaccine.

Introduction

COVID-19 vaccines are widely available in the United States, but portions of the population remain unvaccinated. Weekly vaccinations peaked in April 2021 as vaccine eligibility opened to all adults, and within three months had plateaued at just over half the population greater than 12 years old [1]. According to a study of 39 nationally representative polls taken in the beginning of 2021, approximately one third of the population appeared skeptical or unwilling to take the vaccine, and as of November 2021 vaccination rates in a number of states are below 50% [2]. Most of these polls, as well as the scientific research that has been conducted on vaccine hesitancy, was conducted prior to this pivotal period in April when vaccine supply became available to everyone. Furthermore, the public evaluation of vaccine safety and efficacy should be considered within the empirical context of nearly 200 million individuals who have

been fully vaccinated, the rarity of adverse events, and the apparent effectiveness of the vaccines at preventing COVID-19 hospitalizations and death. The persistence of substantial vaccine reluctance despite the overwhelming evidence of its utility and the ready accessibility to vaccine stocks suggests that there may be cultural, historical, and political cues that shape people's vaccine intentions. Understanding the factors that contribute to such reluctance can help risk communication strategies that promote effective protective behaviors. This is particularly critical given a public health threat that has both individual and population repercussions.

A growing body of literature has determined that vaccine hesitancy is a complex decision-making process influenced by experience, risk perception, culture, confidence in authorities and medicine [3, 4]. Hesitancy varies by convenience, complacency, and confidence, and can vary vaccine to vaccine because of these factors- meaning the level of confidence in vaccine A may be different than vaccine B, as would level of convenience to access the vaccine itself. This means that trends seen related to uptake of existing vaccine such as flu, HPV, MMR, polio, etc. may not be consistent for a new vaccine, and that for each vaccine there is a confluence of factors that will relate to its uptake. Vaccine hesitancy exists on a continuum ranging from overt acceptance to uncertainty, delay, and outright refusal. Several scholars have also found that movement along [up or down] this continuum of hesitancy can be a dynamic process, with individuals' views changing in relation to available and trusted information, social context, and personal beliefs [3].

Prior to the COVID-19 vaccine rollout, researchers investigated the relationship among vaccine hesitancy and economic and demographic factors using traditional surveys or online panels [5–11]. Because of the varying 3Cs depending on the vaccine, there is expected variation in which demographics are most associated with vaccine uptake. While most studies found that Black adults were more likely to be vaccine hesitant than white adults, findings regarding other demographic characteristics were more mixed [2, 12–15]. For instance, Viswanath found that those who were younger were more likely to be vaccine hesitant than those who were older, while Karpman found that those 35–49 were more likely to be vaccine hesitant, compared to both older and younger groups [12, 15]. Findings regarding gender were similarly mixed, with several studies reporting that women were more likely to be vaccine hesitant, others finding the opposite, and some finding no relationship [12, 13]. Findings regarding employment status and education status were similarly mixed [13–15]. Politicization of the pandemic and vaccination is an emerging research topic at the time of this study, with one study finding political party to be predictive of vaccine intention [prior to the vaccine being available] [16].

In addition to demographics, some authors have explored the role of risk perception and exposure as it relates to COVID-19 vaccination intentions. For instance, Viswanath and colleagues examined the relationship between intent to vaccinate and the construct of risk perception, operationalized as susceptibility and severity [15]. While higher perceptions of both susceptibility and severity were associated with intentions to take the vaccine, having someone in the family who contracted COVID-19 was not associated with intent to vaccinate. At the same time, few studies have examined the role of economic impacts of the COVID-19 pandemic on vaccine hesitancy.

The current research on COVID-19 vaccine intentions is mixed in both measurement of the hesitancy outcome and demographic results. Many research studies and polling on COVID-19 vaccination intentions have not fully captured the spectrum of intent, and focused instead on dichotomous outcomes [yes/no], 4 -point Likert scales to capture likelihood in intent, or used an outcome that has three categories [intends to be vaccinated/unsure/does not intend to be vaccinated] [2]. Allowing for a wider range of responses along a continuum of intent/action may provide important insights for increasing vaccination rates. More

importantly, at the time this study was conducted, little research has focused on the period *after* vaccines became available and plentiful while the public health community was mobilized to promote the vaccine. This study is positioned to identify what sociodemographic and pandemic experience variables were associated with the spectrum of vaccine hesitancy using a nationally representative online survey conducted in April 2021.

We specifically sought to answer three research questions in this study:

1. How did vaccination intentions vary on a continuum of vaccine hesitancy in the period of time immediately after vaccines were available?
2. Which sociodemographic factors were associated with being undecided in vaccine intentions?
3. And, what was the relationship between pandemic experience with COVID-19 and level of vaccine hesitancy?

Methods

Study design and data collection

Here we describe our survey, consistent with the Checklist for Reporting Results of Internet E-Surveys (CHERRIES) [17]. An online survey was conducted from a random sample of the Social Science Research Solution [SSRS] Opinion Panel web panelists, representative of U.S. adults age 18 and older who use the internet. The SSRS Opinion Panel uses two sampling methods: 1) an Address-Based Sample [ABS] frame to randomly recruit nationally representative samples, and 2) the SSRS Omnibus survey, a multi-frame random digit dial sample of landlines and cellphones, to recruit harder-to-reach demographic groups. For this study, the SSRS Omnibus survey platform oversampled Blacks, Hispanics, and adults living in rural areas. Survey items were pilot tested with unvaccinated graduate students for cognitive testing prior to administration. The 30-minute pre-tested web survey was emailed to the web panelists with a unique link with a passcode either in English or Spanish based on the respondent's preferred language. As an incentive for participation, a gift card in electronic form was emailed after the survey. To ensure the proper administration of the survey and the content of the questionnaire, a "soft launch" was conducted on April 7, 2021, to a limited number of the respondents. The survey was fully launched to the rest of the sample on April 8, 2021 and completed on April 22, 2021. An email invitation was sent to 6,233 individuals. Approximately 53% replied. Among those who responded, 307 individuals either did not complete the survey or refused to participate. SSRS used "trap" questions as standard quality control checks in their survey., one of the questions asked respondents to *"select the option below that spells out the num*ber *"7"' and* the options given were "1*] Lizard*, *2] Bowl*, *3] Seven*, *4] Ruler*, *5] Lunch*." The respondents who answered three or more of the trap questions incorrectly were removed from the analysis sample. Those whose survey length was less than 5.5 minutes [less than 20% of the average length for the full sample] as well as those who skipped more than 10% of the questions were eliminated from the sample. A total of four completed surveys were removed after applying these cleaning standards. A respondent who did not answer their vaccination status [which was the outcome variable] was also eliminated. Our final sample included 3013 respondents who completed the survey. IRB approval was obtained through the NYU Institutional Review Board and the panelists were provided with a digital consent form at the beginning of the survey.

Outcome variable

Survey respondents were asked whether they have received the COVID-19 vaccine. The response options were 1) fully vaccinated 2) partially vaccinated 3)not yet vaccinated. Those who had not yet been vaccinated were asked about their likelihood of taking the COVID-19 vaccine once becoming eligible. To capture the spectrum of vaccine hesitancy, answer categories included: 1) take it as soon as possible (eager), 2) wait to see how it goes before taking it (wait-and-see), 3) undecided, and 4) will not take the vaccine [refuse]. Fully or partially vaccinated respondents were combined into a single category, resulting in five categories for our dependent variable. For the ease of interpretation, the five categories will hereafter be referred to as vaccinated, eager, wait, undecided, and refuse.

Independent variables

The independent variables included sociodemographic characteristics as well as COVID-19 experience variables.

Sociodemographics. For the sociodemographic characteristics, age [18–29, 30–49, 50–64 and 65 and older], sex [female and male], race and ethnicity [non-Hispanic White, non-Hispanic Black, Hispanic and other], educational attainment [Less than or graduated high school, some college or graduated college, and post graduate or professional degree], annual household income [≤$25,000, $25,001-≤$50,000, $50,001-≤$75,000, $75,001-≤$100,000, ≥$100,001], religion [Protestant, Evangelical Catholic, other and Agnostic/Atheist], living in rural or metropolitan/suburban areas, types of health insurance [private, Medicare, Medicaid, TRICARE/Indian HS/Veteran/Other and uninsured], being a parent [living with children under age 18], and political party affiliation [Democrat, Republican, independent, and don't know] were included.

COVID-19 experience. To understand a respondents experience with COVID-19, we asked whether the respondent had been infected with COVID-19, personally knew someone who died of COVID-19, or was financially impacted by COVID-19 in the form of losing a job, losing income, and/or trouble paying for rent and other necessities. These were each binary indicator variables. Financial severity is an index score of financial hardships including losing a job, losing income, trouble paying for necessities. The minimum is 0, indicating no financial hardship and maximum is 3 indicating hardships in all three areas.

Statistical analysis

Descriptive and bivariate analyses used chi-square tests to evaluate associations between the distributions of the sociodemographic characteristics as well as the COVID-19 experiences with the five different outcome categories. Chi-square tests of independence were conducted between the five outcome groups and predictor variables. We used unadjusted multinomial logistic regression models to estimate the independent effect of each predictor, then built an adjusted multinomial logistic regression model with all the covariates to examine adjusted risk ratios. We also tested a series of models with interaction terms [political party and age, education and race, gender and age/education/race, ethnicity and knowing someone who died of COVID personally, and education and financial hardship]. Although we considered using ordered logistic regression, the data did not meet the proportional odds assumption, nor would such an approach allow us to maintain the fully/partially vaccinated group as a consistent reference group, so we used multinomial logistic modeling. A forest plot was drawn after running the multivariable multinomial regression to assess the effect size of each variable. A multicollinearity test was conducted by examining the conditionings of the indices [using Stata "coldiag2"] and a condition number of 30 or greater was used as a cut-off value [18]. A

Generalized Hosmer-Lemeshow goodness of fit test was used to assess the model fit. Data analysis was performed using Stata version 15 and a p-value of 0.05 was used as a threshold for statistical significance.

Results

Sample characteristics

Table 1 shows the distribution of sociodemographic characteristics as well as the COVID-19 exposure variables by the vaccine acceptance groups. Chi-squared tests show that the distributions of each variable are statistically significantly different by the vaccine acceptance groups and that the five different outcome groups were statistically significantly different from each other in all the variables listed in Table 1. In our sample, more than half of the respondents [59%] were either fully or partially vaccinated at the time of our survey. About 10% of the respondents reported that they were eager to be vaccinated, while the rest of the respondents' [approx. 30%] answers fell along the COVID-19 vaccine hesitancy spectrum. Over 85% of the sample had not had COVID-19, and the majority had not known anyone personally who died of COVID-19 [59.2%]. Over 40% of the respondents indicated that they had experienced financial hardships resulting from the pandemic.

Multinomial logistic regressions

We conducted both unadjusted and adjusted multivariable multinomial regressions to estimate the independent and controlled effect of each independent variable on vaccine acceptance. Our research questions asked [2] which sociodemographics were related to which levels of the hesitancy spectrum and [3] what was the relationship between COVID-19 experience and hesitancy level, so we report these results by hesitancy level below. Results of unadjusted multinomial regressions are shown in Table 2. Below we describe the results of adjusted multinomial regression for each level of the vaccine hesitancy spectrum compared to those that were fully or partially vaccinated as a reference group [Table 3]. Our description focuses on which factors were associated with being more likely to fall in that level of the hesitancy spectrum. Effect size and range of relative risk ratios can be seen in Fig 1.

Eager. Age was a significant predictor in the adjusted multivariable multinomial regression. Those who are younger were more likely than those over 65 to be in the eager group. Female respondents were more likely to be in the eager group compared to Males [aRRR = 0.72, 95%CI:0.55–0.94]. Employment status showed a statistical significance, indicating that those who were unemployed had 1.7 times the risk of being in the eager group compared to the employed [95%CI:1.23–2.35]. The aRRR for urban respondents was 2.06, showing that they were more likely than non-urban dwellers to be in the eager group. When compared to respondents who live in the Northeast region, those who live in the South and the West regions were less likely to be in the eager group [aRRR = 0.55, 95% CI:0.38–0.79 for South region, aRRR = 0.67, 95% CI:0.45–0.98 for North region]. Those without health insurance had a higher risk of being in the eager group [aRRR = 2.43, 95%CI:1.42–4.17] compared to those with private health insurance. The aRRR for being in the eager group was 1.69 [95% CI: 1.25–2.29] for those with independent political affiliation, indicating that they were more likely to be in the eager compared to Democrats. Among the COVID-19 exposure variables, those who had COVID-19 were more likely than the uninfected to be in the eager group [aRRR = 1.47, 95% CI:1.01–2.14].

Wait. Those who are younger were more likely than those over 65 to be in the wait group. Non-Hispanic black and Hispanic respondents were 2.45 and 1.75 times the risk of being in the wait group compared to the non-Hispanic white counterparts, respectively. Those with less

Table 1. Sociodemographic characteristics and COVID risk perception of the samples by vaccination status.

	All [n = 3013]	Fully/partially vaccinated [n = 1779, 59.0%]	Eager-to-take [n = 313, 10.4%]	Wait-and-see [n = 274, 9.1%]	Undecided [n = 310, 10.3%]	Refuse [n = 337, 11.2%]	*p*-value
**Age groups**							<0.001
18–29	474 [15.8%]	205 [11.6%]	84 [27.0%]	73 [26.7%]	56 [18.2%]	56 [16.7%]	
30–49	1112 [37.1%]	527 [29.8%]	144 [46.3%]	134 [49.1%]	146 [47.4%]	161 [47.9]	
50–64	786 [26.2%]	508 [28.7%]	66 [21.2%]	49 [18.0%]	82 [26.6%]	81 [24.1%]	
65+	625 [20.9%]	529 [29.9%]	17 [5.5%]	17 [6.2%]	24 [7.8%]	38 [11.3%]	
**Gender**							<0.001
Female	1370 [45.7%]	845 [47.8%]	163 [52.8%]	119 [43.8%]	122 [39.4%]	121 [36.0%]	
Male	1626 [54.3%]	924 [52.2%]	146 [47.3%]	153 [56.3%]	188 [60.7%]	215 [64.0%]	
**Race/Ethnicity**							<0.001
White Non-Hispanic	1684 [56.4%]	1053 [59.7%]	154 [49.5%]	123 [45.2%]	163 [53.3%]	191 [57.0%]	
Black Non-Hispanic	569 [19.0%]	313 [17.7%]	41 [13.2%]	74 [27.2%]	68 [22.2%]	73 [21.8%]	
Hispanic	531 [17.8%]	260 [14.7%]	77 [24.8%]	64 [23.5%]	67 [21.9%]	63 [18.8%]	
Other	204 [6.8%]	138 [7.8%]	39 [12.5%]	11 [4.0%]	8 [2.6%]	8 [2.4%]	
**Education**							<0.001
Less than/graduated high school	614 [20.4%]	256 [14.4%]	85 [27.2%]	62 [22.6%]	100 [32.4%]	111 [32.9%]	
Some college or graduated college	1551 [51.5%]	885 [49.8%]	151 [48.2%]	165 [60.2%]	171 [55.3%]	179 [53.1%]	
Post-graduate/professional	847 [28.1%]	638 [35.9%]	77 [24.6%]	47 [17.2%]	38 [12.3%]	47 [14.0%]	
**Employment status**							0.045
Unemployed	1071 [35.6%]	663 [37.3%]	116 [37.1%]	85 [31.0%]	93 [30.0%]	114 [33.8%]	
Employed [full or part time]	1940 [64.4%]	1114 [62.7%]	197 [62.9%]	189 [69.0%]	217 [70.0%]	223 [66.2%]	
**Annual Income**							<0.001
Less than $25,000	515 [17.1%]	207 [11.6%]	68 [21.8%]	69 [25.3%]	78 [25.2%]	93 [27.6%]	
$25,000 to less than $50,000	656 [21.8%]	348 [19.6%]	80 [25.6%]	69 [25.3%]	83 [26.8%]	76 [22.6%]	
$50,000 to less than $75,000	573 [19.0%]	361 [20.3%]	48 [15.4%]	46 [16.9%]	62 [20.0%]	56 [16.6%]	
$75,000 to less than$100,000	464 [15.4%]	294 [16.5%]	38 [12.2%]	36 [13.2%]	49 [15.8%]	47 [14.0%]	
$100,000 or more	803 [26.7%]	569 [32.0%]	78 [25.0%]	53 [19.4%]	38 [12.3%]	65 [19.3%]	
**Religion**							<0.001
Protestant	650 [21.6%]	368 [20.8%]	45 [14.4%]	71 [26.0%]	75 [24.2%]	91 [27.1%]	
Evangelical	193 [6.4%]	91 [5.1%]	13 [4.2%]	28 [10.3%]	29 [9.4%]	32 [9.5%]	
Catholic, Roman Catholic	633 [21.1%]	399 [22.5%]	74 [23.6%]	41 [15.0%]	64 [20.7%]	55 [16.4%]	
Other	635 [21.1%]	398 [22.5%]	52 [16.6%]	52 [19.1%]	59 [19.0%]	74 [22.0%]	
Nothing in particular/Atheist/ Agnostic	894 [29.8%]	517 [29.2%]	129 [41.2%]	81 [29.7%]	83 [26.8%]	84 [25.0%]	
**Metro Status**							<0.001
Non-metro	534 [17.9%]	304 [17.3%]	31 [10.0%]	41 [15.4%]	64 [20.9%]	94 [28.2%]	
Metro	2444 [82.1%]	1456 [82.7%]	280 [90.0%]	226 [84.6%]	243 [79.2%]	239 [71.8%]	
**Census region**							<0.001
North East	546 [18.3%]	335 [19.0%]	80 [25.7%]	45 [16.7%]	43 [14.0%]	43 [12.8%]	
North Central	625 [20.9%]	366 [20.7%]	62 [19.9%]	58 [21.6%]	60 [19.5%]	79 [23.6%]	

(*Continued*)

Table 1. (Continued)

	All [n = 3013]	Fully/partially vaccinated [n = 1779, 59.0%]	Eager-to-take [n = 313, 10.4%]	Wait-and-see [n = 274, 9.1%]	Undecided [n = 310, 10.3%]	Refuse [n = 337, 11.2%]	*p*-value
South	1158 [38.7%]	664 [37.6%]	96 [30.9%]	121 [45.0%]	132 [42.9%]	145 [43.3%]	
West	661 [22.1%]	402 [22.8%]	73 [23.5%]	45 [16.7%]	73 [23.7%]	68 [20.3%]	
**Health Insurance**							<0.001
Private	1566 [43.8%]	946 [53.2%]	173 [55.3%]	144 [52.6%]	159 [51.3%]	144 [42.7%]	
Medicare	666 [24.1%]	530 [29.8%]	24 [7.7%]	28 [10.2%]	41 [13.2%]	43[12.8%]	
Medicaid	400 [17.8%]	156 [8.8%]	60 [19.2%]	56 [20.4%]	53 [17.1%]	75 [22.3%]	
TRICARE/VA/Indian Health Service/Other	187 [6.2%]	101 [5.7%]	20 [6.4%]	18 [6.6%]	21 [6.8%]	27 [8.0%]	
Uninsured	193 [6.4%]	45 [2.5%]	36 [11.5%]	28 [10.2%]	36 [11.6%]	48 [14.2%]	
**Parent**							<0.001
No	2109 [70.4%]	1375 [77.6%]	205 [65.7%]	163 [60.4%]	189 [62.0%]	177 [52.7%]	
Yes	886 [29.6%]	397 [22.4%]	107 [34.3%]	107 [39.6%]	116 [38.0%]	159 [47.3%]	
**Political Party**							<0.001
Republican	713 [23.7%]	352 [19.8%]	41 [13.1%]	78 [28.5%]	106 [34.2%]	136 [40.4%]	
Democrat	1162 [38.6%]	832 [46.8%]	124 [39.6%]	75 [27.4%]	74 [23.9%]	57 [16.9%]	
Independent	996 [33.1%]	528 [29.7%]	133 [42.5%]	106 [38.7%]	111 [35.8%]	118 [35.0%]	
Other	142 [4.7%]	67 [3.8%]	15 [4.8%]	15 [5.5%]	19 [6.1%]	26 [7.7%]	
**Have you had COVID-19**							<0.001
No	2606 [86.5%]	1580 [88.8%]	265 [84.7%]	232 4.7%]	259 [83.6%]	270 [80.1%]	
Yes	407 [13.5%]	199 [11.2%]	48 [15.3%]	42 [15.3%]	51 [16.5%]	67 [19.9%]	
**Personally know anyone who died of COVID-19**							<0.001
No	1785 [59.2%]	981 [55.1%]	199 [63.6%]	178 [65.0%]	195 [62.9%]	232 [68.8%]	
Yes	1228 [40.8%]	798 [44.9%]	114 [36.4%]	96 [35.0%]	115 [37.1%]	105 [31.2%]	
**Financial severity***							<0.001
0	1860 [61.7%]	1230 [69.1%]	172 [55.0%]	139 [50.7%]	153 [49.5%]	166 [49.3%]	
1	844 [28.0%]	422 [23.7%]	105 [33.6%]	85 [31.0%]	107 [34.5%]	125 [37.1%]	
2	186 [6.2%]	83 [4.7%]	27 [8.6%]	30 [11.0%]	22 [7.1%]	24 [7.1%]	
3	123 [4.1%]	44 [2.5%]	9 [2.9%]	20 [7.3%]	28 [9.0%]	22 [6.5%]	

* Financial severity is an index score for losing job, losing income, and trouble paying rent or other necessities in a scale of 0 to 3. 0 indicates no financial difficulty and 3 indicates financial difficulties in all 3 areas.

https://doi.org/10.1371/journal.pone.0267734.t001

education were more likely than those with post-graduate or professional degrees to be in the wait group [aRRR = 2.40, 95%CI: 1.49–3.88 for high school graduates or less, RRR = 1.75, 95% CI: 1.20–2.55 for college graduates or less]. Compared to those in the highest income group, those who belonged to the lowest income group had 1.81 times the risk of being in the wait group [95%CI: 1.05–3.12] compared to the respondents in the highest income group. Respondents who identified their political affiliation as Republican or Independent were more likely

Table 2. Unadjusted multinomial regression of unvaccinated groups compared to fully/partially vaccinated group as a reference category.

	Relative risk of each unvaccinated group compared to fully/partially vaccinated group [RRR, 95%CI]			
	**Eager-to-take**	**Wait-and-see**	**Undecided**	**Refuse**
**Demographic characteristics**				
**Age groups**				
18–29	12.75 [7.39, 22.00] *	11.08 [6.38, 19.24] *	6.02 [3.64, 9.97] *	3.80 [2.44, 5.92] *
30–49	8.50 [5.07, 14.26] *	7.91 [4.71, 13.29] *	6.11 [3.90, 9.56] *	4.25 [2.93, 6.18] *
50–64	4.04 [2.34, 6.98] *	3.00 [1.71, 5.28] *	3.56 [2.22, 5.70] *	2.22 [1.48, 3.33] *
65+	-	-	-	-
**Gender**				
Female	-	-	-	-
Male	0.82 [0.64, 1.04]	1.18 [0.91, 1.52]	1.41 [1.10, 1.80] *	1.62 [1.28, 2.07] *
**Race/Ethnicity**				
White Non-Hispanic	-	-	-	-
Black Non-Hispanic	0.90 [0.62, 1.29]	2.02 [1.48, 2.77] *	1.40 [1.03, 1.91] *	1.29 [0.95, 1.73]
Hispanic	2.02 [1.49, 2.75] *	2.11 [1.51, 2.93] *	1.66 [1.21, 2.28] *	1.34 [0.97, 1.83]
Other	1.93 [1.30, 2.86] *	0.68 [0.36, 1.30]	0.37 [0.18, 0.78] *	0.32 [0.15, 0.66] *
**Education**				
Less than/graduated high school	2.75 [1.96, 3.87] *	3.29 [2.19, 4.93] *	6.56 [3.49, 9.79] *	5.89 [4.06, 8.53] *
Some college or graduated college	1.41 [1.05, 1.89] *	2.53 [1.80, 3.55] *	3.24 [2.25, 4.68] *	2.75 [1.96, 3.85] *
Post-graduate/professional	-	-	-	-
**Employment status**				
Unemployed	0.99 [0.77, 1.27]	0.76 [0.57, 0.99] *	0.72 [0.55, 0.94] *	0.86 [0.67, 1.10]
Employed [full or part time]	-	-	-	-
**Annual Income**				
Less than $25,000	2.40 [1.67, 3.44] *	3.58 [2.42, 5.29] *	5.64 [3.71, 8.58] *	3.93 [2.76, 5.61] *
$25,000 to less than $50,000	1.68 [1.19, 2.35] *	2.13 [1.45, 3.12] *	3.57 [2.38, 5.36] *	1.91 [1.33, 2.73] *
$50,000 to less than $75,000	0.97 [0.66, 1.42]	1.37 [0.90, 2.07]	2.57 [1.68, 3.93] *	1.36 [0.93, 1.99]
$75,000 to less than $100,000	0.94 [0.62, 1.42]	1.31 [0.84, 2.05]	2.50 [1.60, 3.90] *	1.40 [0.94, 2.09]
$100,000 or more	-	-	-	-
**Religion**				
Protestant	-	-	-	-
Evangelical	1.17 [0.60, 2.26]	1.59 [0.97, 2.61]	1.56 [0.96, 2.54]	1.42 [0.89, 2.26]
Catholic, Roman Catholic	1.52 [1.02, 2.26] *	0.53 [0.35, 0.80] *	0.79 [0.55, 1.13]	0.56 [0.39, 0.80] *
Other	1.07 [0.70, 1.63]	0.68 [0.46, 1.00]	0.73 [0.50, 1.05]	0.75 [0.54, 1.05]
Nothing in particular/Atheist/Agnostic	2.04 [1.42, 2.94] *	0.81 [0.57, 1.15]	0.79 [0.56, 1.11]	0.66 [0.47, 0.91] *
**Metro Status**				
Non-metro/rural	-	-	-	-
Metro	1.89 [1.28, 2.79] *	1.15 [0.81, 1.64]	0.79 [0.59, 1.07]	0.53 [0.41, 0.69] *
**Census region**				
North East	-	-	-	-
North Central	0.71 [0.49, 1.02]	1.18 [0.78, 1.79]	1.28 [0.84, 1.94]	1.68 [1.13, 2.51] *
South	0.61 [0.44, 0.83] *	1.36 [0.94, 1.96]	1.55 [1.07, 2.24] *	1.70 [1.18, 2.45] *
West	0.76 [0.54, 1.08]	0.83 [0.54, 1.29]	1.41 [0.94, 2.12]	1.32 [0.88, 1.98]
**Health insurance type**				
Private	-	-	-	-
Medicare	0.25 [0.16, 0.38] *	0.35 [0.23, 0.53] *	0.46 [0.32, 0.66] *	0.53 [0.37, 0.76] *
Medicaid	2.10 [1.50, 2.95] *	2.36 [1.66, 3.35] *	2.02 [1.42, 2.88] *	3.16 [2.28, 4.38] *
TRICARE/ Veterans/Indian HS/ Other	1.08 [0.65, 1.80]	1.17 [0.69, 1.99]	1.24 [0.75, 2.04]	1.76 [1.11, 2.78]

(*Continued*)

Table 2. (Continued)

	Relative risk of each unvaccinated group compared to fully/partially vaccinated group [RRR, 95%CI]			
	**Eager-to-take**	**Wait-and-see**	**Undecided**	**Refuse**
Uninsured	4.37 [2.74, 6.98] *	4.09 [2.47, 6.76] *	4.76 [2.98, 7.61] *	7.01 [4.50, 10.91] *
**Parent**				
No	-	-	-	-
Yes	1.81 [1.40, 2.34] *	2.27 [1.74, 2.97] *	2.13 [1.64, 2.75] *	3.11 [2.44, 3.96] *
**Political party**				
Democrat	-	-	-	-
Republican	0.78 [0.54, 1.14]	2.46 [1.75, 3.46] *	3.39 [2.45, 4.67] *	5.64 [4.04, 7.87] *
Independent	1.69 [1.29, 2.21] *	2.23 [1.62, 3.05] *	2.36 [1.73, 3.23] *	3.26 [2.33, 4.56] *
Other, don't know, refused	1.50 [0.83, 2.71]	2.48 [1.35, 4.56] *	3.19 [1.82, 5.59] *	5.66 [3.34, 9.59] *
**COVID exposure/experience**				
**Had COVID-19**				
No	-	-	-	-
Yes	1.44 [1.02, 2.02] *	1.44 [1.00, 2.06]	1.56 [1.12, 2.18] *	1.97 [1.45, 2.67] *
**Personally know anyone died of COVID-19**				
No	-	-	-	-
Yes	0.70 [0.55, 0.90] *	0.66 [0.51, 0.86] *	0.72 [0.57, 0.93] *	0.56 [0.43, 0.71] *
**Financial severity [mean, SD]** **	1.40 [1.20, 1.63] *	1.72 [1.48, 1.99] *	1.74 [1.51, 2.00] *	1.64 [1.42, 1.88] *

* p-value <0.05

** Financial severity is an index score of financial hardships including losing a job, losing income, trouble paying for necessities. The minimum is 0, indicating no financial hardship and maximum is 3 indicating hardships in all three areas.

https://doi.org/10.1371/journal.pone.0267734.t002

than Democrats to be in the wait [aRRR = 5.53, 95% CI:3.59–8.53 for Republicans, RRR = 3.04, 95% CI: 2.12–4.37 for Independent]. The higher the financial severity, the higher the risk of being in the wait group [aRRR = 1.23, 95%CI: 1.06–1.48] group.

Undecided. Compared to 65 and older respondents, those who were younger were more likely to be undecided. Compared to non-Hispanic whites, non-Hispanic black respondents had 2.18 times the risk of being undecided [95% CI: 1.45–3.27]. Those who had less education were more likely than those who had post-graduate or professional degrees to be undecided [aRRR = 4.05, 95% CI: 2.54–6.44 for high school graduates or less, aRRR = 2.12, 95% CI: 1.42–3.17]. Similarly, those in lower-income level groups had a higher risk than those with the highest income group of being in the undecided group [aRRRs 3.17–2.04]. The respondents with no health insurance had 1.96 times the risk of being in the undecided group compared to those with private health insurance [95% CI:1.13–3.40]. Compared to Democrats, all the other groups of political affiliation i.e., Republican, Independent, and other/don't know/refused respondents were more likely to be in the undecided group and that risk was highest among the Republicans [aRRR = 7.77, 95% CI:4.40–10.1]. In terms of COVID-19 exposure, if respondents personally knew someone who died of COVID-19, they were less likely to be undecided than those who did not know someone who died of COVID-1 [aRRR = 0.64, 95% CI: 0.49–0.85]. In contrast, those with severe financial hardship had 1.25 times the risk of being undecided compared to those without any financial hardship [aRRR = 1.25, 95% CI: 1.06–1.48].

Refuse. Compared to 65 and older, the respondents in the two youngest age groups had a higher risk of being in the refuse group. Males had 1.46 times the risk of refusing the vaccine compared to females [95%CI: 1.10–1.94]. Non-Hispanic blacks had 2.63 times the risk of being in the refuse group compared to Non-Hispanic whites [95% CI:1.74–3.95]. Following

Table 3. Adjusted multivariable multinomial regression of unvaccinated groups compared to fully/partially vaccinated group as a reference category [n = 2899].

	Relative risk of each unvaccinated group compared to fully/partially vaccinated group [RRR, 95%CI]			
	Eager-to-take	Wait-and-see	Undecided	Refuse
	n = 302	n = 256	n = 296	n = 328
**Demographic characteristics**				
**Age groups**				
18–29	6.62 [3.05, 14.39] *	9.01 [4.04, 20.1] *	5.63 [2.69, 11.80] *	2.26 [1.13, 4.53] *
30–49	5.79 [2.71, 12.37] *	7.57 [3.47, 16.5] *	6.90 [3.42, 13.88] *	2.66 [1.39, 5.08] *
50–64	2.84 [1.35, 5.94] *	2.63 [1.23, 5.63] *	3.57 [1.84, 6.94] *	1.61 [0.87, 2.96]
65+	-	-	-	-
**Gender**				
Female	-	-	-	-
Male	0.72 [0.55, 0.94] *	1.01 [0.75, 1.36]	1.15 [0.87, 1.52] *	1.46 [1.10, 1.94] *
**Race/Ethnicity**				
White Non-Hispanic	-	-	-	-
Black Non-Hispanic	0.84 [0.54, 1.30]	2.45 [1.61, 3.74] *	2.18 [1.45, 3.27] *	2.63 [1.74, 3.95] *
Hispanic	1.07 [0.73, 1.57]	1.72 [1.14, 2.60] *	1.14 [0.76, 1.72]	1.37 [0.91, 2.07]
Other	1.29 [0.82, 2.04]	0.58 [0.28, 1.23]	0.49 [0.18, 0.78]	0.48 [0.22, 1.05]
**Education**				
Less than/graduated high school	2.20 [1.45, 3.34] *	2.40 [1.49, 3.88] *	4.05 [2.54, 6.44] *	3.54 [2.28, 5.51] *
Some college or graduated college	1.11 [0.80, 1.54]	1.75 [1.20, 2.55] *	2.12 [1.42, 3.17] *	1.74 [1.19, 2.53] *
Post-graduate/professional	-	-	-	-
**Employment status**				
Unemployed	1.70 [1.23, 2.35] *	1.12 [0.78, 1.61]	0.85 [0.61, 1.20]	0.89 [0.64, 1.24]
Employed [full or part time]	-	-	-	-
**Annual Income**				
Less than $25,000	1.55 [0.93, 2.56]	1.81 [1.05, 3.12] *	3.37 [1.95, 5.84] *	1.58 [0.95, 2.62]
$25,000 to less than $50,000	1.50 [0.98, 2.30]	1.45 [091, 2.31]	2.71 [1.68, 4.37] *	1.07 [0.68, 1.69]
$50,000 to less than $75,000	1.02 [0.67, 1.55]	1.10 [0.69, 1.74]	2.17 [1.36, 3.45] *	0.97 [0.63, 1.50]
$75,000 to less than $100,000	1.00 [0.64, 1.56]	1.11 [0.68, 1.82]	2.04 [1.25, 3.32] *	1.08 [0.69, 1.70]
$100,000 or more	-	-	-	-
**Religion**				
Protestant	-	-	-	-
Evangelical	0.83 [0.40, 1.69]	1.30 [0.74, 2.26]	1.29 [0.75, 2.24]	1.08 [0.64, 1.83]
Catholic, Roman Catholic	1.02 [0.64, 1.61]	0.57 [0.35, 0.91] *	0.91 [0.59, 1.41]	0.65 [0.42, 1.01]
Other	0.83 [0.52, 1.32]	0.77 [0.49, 1.19]	0.87 [0.57, 1.31]	0.88 [0.59, 1.30]
Nothing in particular/Atheist/Agnostic	1.31 [0.87, 2.00]	0.93 [0.62, 1.40]	0.98 [0.66, 1.46]	0.91 [0.61, 1.34]
**Metro Status**				
Non-metro/rural	-	-	-	-
Metro	2.06 [1.32, 3.21] *	1.22 [0.82, 1.83]	0.95 [0.67, 1.35]	0.71 [0.51, 0.98] *
**Census region**				
North East	-	-	-	-
North Central	0.68 [0.45, 1.01]	0.93 [0.60, 1.47]	0.95 [0.60, 1.50]	1.20 [0.77, 1.88]
South	0.55 [0.38, 0.79] *	0.86 [0.57, 1.29]	1.04 [0.69, 1.57]	1.02 [0.68, 1.55]
West	0.67 [0.45, 0.98] *	0.59 [0.36, 0.95] *	1.35 [0.87, 2.12]	1.11 [0.70, 1.76]
**Health insurance type**				
Private	-	-	-	-
Medicare	0.48 [0.24, 0.93] *	0.93 [0.49, 1.79]	1.10 [0.61, 1.98]	1.03 [0.56, 1.87]
Medicaid	1.13 [0.73, 1.76]	1.18 [0.74, 1.88]	0.95 [0.60, 1.49]	1.84 [1.19, 2.86] *
TRICARE/ Veterans/Indian HS/ Other	0.97 [0.65, 1.66]	0.88 [0.49, 1.55]	0.84 [0.49, 1.46]	1.36 [0.81, 2.30]

(*Continued*)

Table 3. (Continued)

	Relative risk of each unvaccinated group compared to fully/partially vaccinated group [RRR, 95%CI]			
	**Eager-to-take**	**Wait-and-see**	**Undecided**	**Refuse**
	**n = 302**	**n = 256**	**n = 296**	**n = 328**
Uninsured	2.43 [1.42, 4.17] *	1.35 [0.73, 2.52]	1.96 [1.13, 3.40] *	4.20 [2.46, 7.18] *
**Parent**				
No	-	-	-	-
Yes	1.13 [0.82, 1.55]	1.22 [0.88, 1.70]	1.23 [0.89, 1.70]	1.92 [1.40, 2.64] *
**Political party**				
Democrat	-	-	-	-
Republican	1.20 [0.78, 1.85]	5.53 [3.59, 8.53] *	6.66 [4.40, 10.07] *	11.06 [7.21, 16.96] *
Independent	1.69 [1.25, 2.29] *	3.04 [2.12, 4.37] *	3.02 [2.11, 4.33] *	4.30 [2.93, 6.30] *
Other, don't know, refused	1.16 [0.61, 2.22]	2.02 [0.98, 4.15]	3.16 [1.71, 5.82] *	5.48 [3.05, 9.85] *
**COVID exposure/experience**				
**Had COVID-19**				
No	-	-	-	-
Yes	1.47 [1.01, 2.14] *	1.26 [0.84, 1.87]	1.29 [0.89, 1.88]	1.59 [1.11, 2.26] *
**Personally know anyone died of COVID-19**				
No	-	-	-	-
Yes	0.75 [0.57, 0.99] *	0.65 [0.48, 0.87] *	0.64 [0.49, 0.85] *	0.49 [0.37, 0.66] *
**Financial severity [mean, SD]** **	1.01 [0.84, 1.20]	1.23 [1.03, 1.46] *	1.25 [1.06, 1.48] *	1.21 [1.02, 1.44] *

* p-value <0.05

** Financial severity is an index score of financial hardships including losing a job, losing income, trouble paying for necessities. The minimum is 0, indicating no financial hardship and maximum is 3 indicating hardships in all three areas.

https://doi.org/10.1371/journal.pone.0267734.t003

the same trend, those with less education had a higher risk than those with post-graduate or professional degrees to be in the refusal group [aRRR = 3.54, 95% CI:2.28–5.51 for high school graduates or less, RRR = 1.74, 95% CI: 1.19–2.53]. Those who have Medicaid or were uninsured were more likely than those with private health insurance to be in the refuse group [aRRR = 1.84, 95% CI: 1.19–2.86 for Medicaid, aRRR = 4.20, 95% CI: 2.46–7.18 for uninsured]. Compared to Democrats, all other political affiliations were more likely to refuse the vaccine, Republicans showing the highest risk [aRRR = 11.06, 95% CI: 7.21–17.0]. All of the COVID-19 exposure variables were statistically significant factors in predicting the risk of being in the refuse groups. Those who have had COVID-19 infection and those with a severe financial hardship were more likely than those who did not have COVID-19 infection and financial hardship to refuse the vaccine [aRRR = 1.59, 95% CI: 1.11–2.26 for having had COVID-19, aRRR = 1.21, 95% CI: 1.02–1.44 for financial hardship] while those who personally knew someone who died of COVID-19 were less likely than those who did not know someone who died of COVID-19 to be refusers [aRRR = 0.49, 95% CI: 0.37–0.66].

The multivariable multinomial regression models with interactions terms between political party and age, education and race, gender and age, gender and education, gender and race, ethnicity and knowing someone who died of COVID-19, as well as education and severity in financial hardship, were not statistically significant.

Discussion

In this study we explored three research questions. Below we discuss our results in the context of these three questions.

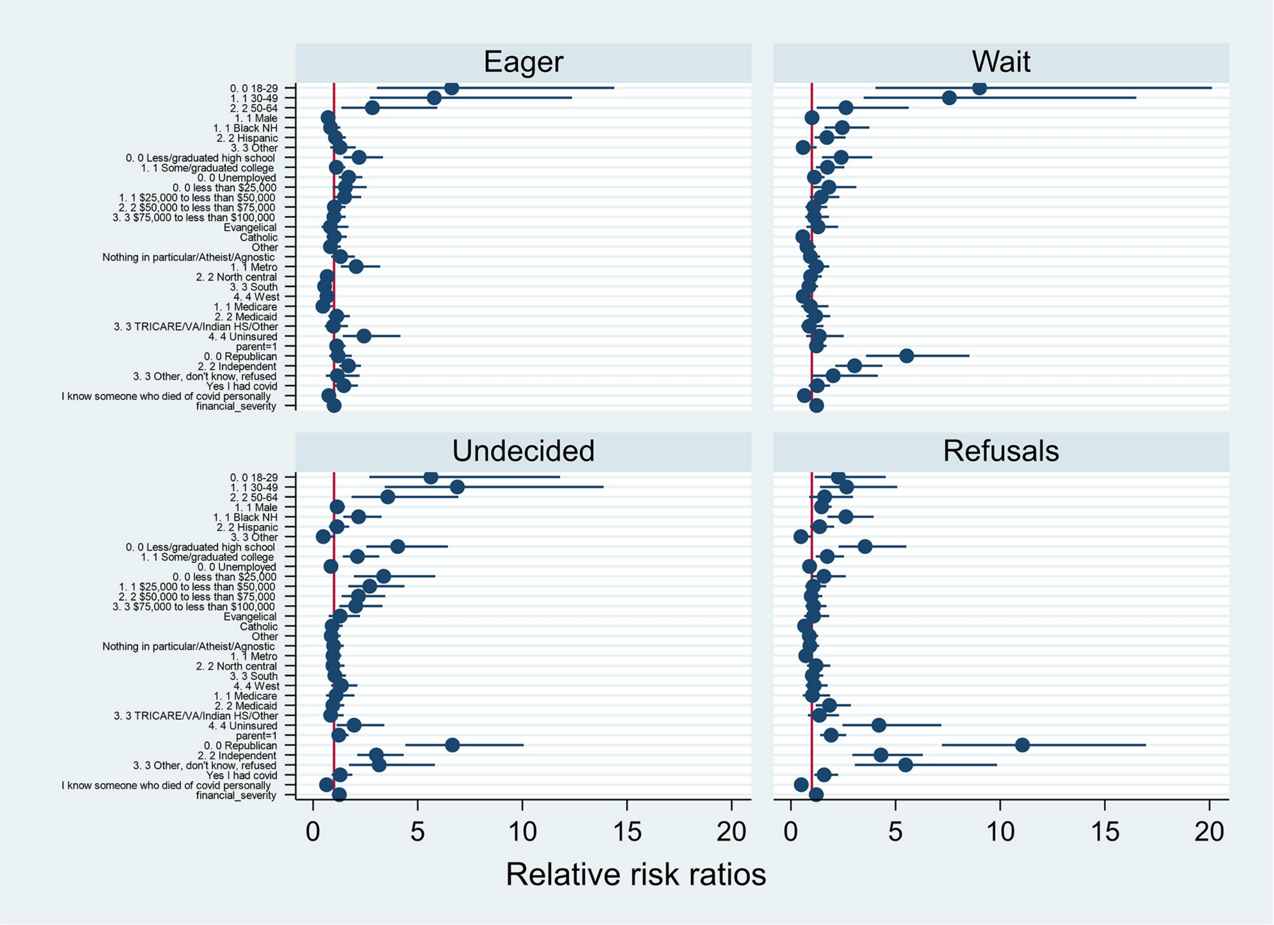

Fig 1.

https://doi.org/10.1371/journal.pone.0267734.g001

How did vaccination intentions vary on a continuum of vaccine hesitancy in the period of time immediately after vaccines were available?

As vaccination opened to the adult public, approximately 40% of our sample was unvaccinated. Those who were unvaccinated fell across four categories- eager to take the vaccine, waiting to see how it goes before deciding to take the vaccine, undecided, and refusing to take the vaccine. The proportion of our sample that was vaccinated was similar to representative national surveys conducted at similar points in time [19].

Which sociodemographic factors were associated with being undecided in vaccine intentions?

Older adults and healthcare workers were among the first vaccine priority groups for vaccine eligibility, meaning they are more likely to be overrepresented in the fully vaccinated part of our sample. Therefore, it is unsurprising that younger ages predicted all categories along the spectrum. As visualized in Fig 1, age had a large effect on being eager to take the vaccine or waiting to see, possibly because for younger age groups eligibility was more recent, and there were evolving social norms among peers. Similarly, lower educational attainment was associated with hesitancy, which in part may be due to the more highly educated healthcare workers

being more likely to be vaccinated [20]. Educational attainment may also be inversely associated with hesitancy due to different levels of understanding of both the threat and vaccine [21, 22]. Lower educational attainment has been found to be an enduring social vulnerability for vaccine hesitancy in the United States [23].

Comparing the demographic differences by hesitancy status, some of the results reported here suggest that hesitancy about the COVID-19 vaccine may be different than vaccine hesitancy for other adult vaccines. For instance, Okoli's study of adult vaccine hesitancy for the flu vaccine found that younger ages were more vaccine hesitant, but several other studies did not [24–26]. Findings regarding gender are similarly mixed. However, numerous studies have found that Blacks have higher odds of vaccine hesitancy than do Whites, as our study confirms here [2, 12–15]. In our study, income was a monotonic and significant predictor for being undecided about getting the vaccine.

Numerous reports in the media and select studies have highlighted the role of political party membership in predicting vaccine hesitancy, and our results confirm these reports [16, 27]. According to the Kaiser Family Foundation [KFF] Vaccine Monitor from November 2021, individuals identifying as Republicans represent the large majority of those who are unvaccinated, in which political affiliation is stronger predictor in comparison to demographic factors. For the "wait and see" group, the undecided group, and those who would refuse the vaccine, being anything other than a Democrat was predictive of higher odds of belonging to those three groups. Only Independents had higher odds than Democrats of being eager to receive the vaccine. The magnitude of the effect size of political party on outright refusal was particularly large. The politicization of the COVID-19 vaccine reflects an emerging trend in vaccination efforts, building on the pushback toward HPV vaccination, and more recently to measles outbreaks [28, 29]. Politicization is associated with decreased support for vaccine mandates, as well as decreased confidence in both doctors and government [30]. The consequences of politicizing COVID-19 and the COVID-19 vaccine, may have longer term consequences on trust in health and governmental workers that extend beyond vaccine uptake. Our analyses shows that even in April 2021, there were effects of political affiliation on vaccine uptake, and efforts, even early on in vaccine rollout [and future public health campaigns], need to be made to identify the communication channels that resonate with different political parties, both among traditional and social media, and key communicators who can act as vaccine ambassadors on these channels and to these groups.

Our analysis posited differences in the association between demographic characteristics and COVID-19 exposures and impact and levels of vaccine hesitancy. We interpreted agreement with the phrase "I will wait to see how it goes before taking the vaccine" [Wait-and-see] as someone who was looking to "see" how something goes before taking the vaccine, with an underlying implication of looking for some event or non-event related to the vaccine. For those who indicated that they were "undecided" about taking the vaccine, we hypothesized that their willingness to take the vaccine would be less than those who indicated they would wait and see, given there was no such acknowledgement of seeking an event or non-event, and expected their reluctance to be more diffuse. This distinction can only be confirmed in future analyses that would explore which group was more likely to be refusers or vaccinators, which are planned by the research team. However, our bivariate analyses reveal differences between these two groups. However, although the results from the multinomial logit models show differences in magnitude for the association between demographics and these outcomes, the only demographic variable that distinguished these groups was Hispanic ethnicity, such that compared to Whites, Hispanics were 73% more likely to indicate that they would "wait and see" before getting the vaccine [RRR = 1.73, 95% CI: 1.14–2.60]. More research is needed to

determine what differences in attitudes, beliefs, and experiences related to vaccines and COVID-19 may distinguish this group.

What was the relationship between risk perception and experience with COVID-19 and level of vaccine hesitancy?

Our findings depict the toll that the pandemic has taken: 41% of our sample knew someone who died of COVID-19, and 38% had suffered some serious financial hardship they attributed to the pandemic. Yet, while we had hypothesized that these experiences would increase the odds of wanting to get the vaccine, we also found that these experiences increased the odds of being in all other categories compared to the fully vaccinated, suggesting that this experience did not necessarily heighten one's willingness to get the vaccine, all else equal.

Similarly, having experienced severe financial hardships resulting from the vaccine was predictive of being in the wait-and-see, undecided, and refuse groups. One hypothesis is that people experiencing these hardships blame government for the mitigation strategies employed for the pandemic [mask-wearing, social distancing, closing bars and restaurants, etc.] and the economic limitations associated with those policies and therefore are less keen on vaccination advice promoted by government officials [31]. As the economic consequences of the pandemic continue to be seen, this may help explain some vaccination holdout. Public health risk communicators therefore must balance the need to promote community mitigation with vaccine promotion.

Our finding that having had COVID-19 increased the relative risk of being both eager to get the vaccine or refusing the vaccine confirms the ways in which these experiences are not uniform and can lead to different behavioral intentions. Perceived threat severity and familiarity typically increase perceived risk and risk is associated with higher behavioral intentions [32]. Compared to prior threats such as Zika and Ebola, vaccine intention and uptake is higher for COVID-19, likely due to the realized threat of COVID-19 in the United States and the severe impact of the virus. However, in both of those outbreaks, pandemic status was not reached, meaning the severity of the threat was less, and vaccine intentions were hypothetical. According to the CDC, flu vaccine uptake typically hovers around 40–50% annually, potentially due to lack of perceived risk for the virus among a large subset of the population. Therefore, more research is needed to understand the actual COVID-19 experience, and if having a more mild case of the virus decreased the perception of the threat and increased apathy towards a vaccine. It may also be that some individuals who had the virus may perceive their natural immunity to be sufficient and that the vaccine is not necessary [33, 34].

This study has its limitations. Our sample differs from the adult population in several ways, as do most online survey panels. We have described the data checks that were embedded by in our survey. Our study is cross-sectional, and thus cannot make claims about causality. Finally, our survey relies on self-report, and focuses on intentions, not behaviors, which may change. At the same time, it offers a snapshot of the US public's intentions just as all adults became eligible for the vaccine, such that intentions could be quickly realized.

Conclusion

Prior research has found individuals' relative position on the hesitancy spectrum is dynamic rather than static [35]. One challenge of identifying the role of COVID-19 experience on vaccine hesitancy is the continuously evolving nature of the lived COVID-19 experience. As research is being conducted, new events are happening in individual's lives that shape perception of the vaccine. Research studies offer a snapshot in time of the relationship between a person's characteristic and experience and their vaccine intentions or behavior. The research

presented here is intended to add perspective on the relationship between sociodemoraphics and COVID-19 experience at the point in time in which vaccines became available to the entire adult US population. This critical moment is the point in which adults were initially faced with a vaccine choice and balanced their experience with the promotion of this intervention. These results can help to inform which demographic groups and which experiences were associated with intentions at that time, and how intentions then evolved since.

Supporting information

S1 Table. Unadjusted multinomial regression of unvaccinated groups compared to fully/ partially vaccinated group as a reference category.
(DOCX)

Author Contributions

Conceptualization: Rachael Piltch-Loeb, Diana R. Silver.

Formal analysis: Yeerae Kim.

Investigation: Rachael Piltch-Loeb, Diana R. Silver, David M. Abramson.

Writing – original draft: Rachael Piltch-Loeb, Yeerae Kim.

Writing – review & editing: Rachael Piltch-Loeb, Diana R. Silver, Hope Norris, Elizabeth McNeill, David M. Abramson.

References

1. CDC. Trends in Number of COVID-19 Vaccinations in the US [Internet]. Atlanta, GA: Centers for Disease Control and Prevention; 2021 [updated 2021 Aug 4; cited 2021 Aug 3]. https://covid.cdc.gov/covid-data-tracker/#vaccination-trends.
2. SteelFisher GK, Blendon RJ, Caporello H. An Uncertain Public—Encouraging Acceptance of Covid-19 Vaccines. N Engl J Med. 2021; 384[16]:1483–7. https://doi.org/10.1056/NEJMp2100351 PMID: 33657291
3. WHO. Global Vaccine Action Plan [Internet]. Geneva: World Health Organization; 2011 [cited 2021 August 3]. https://www.who.int/teams/immunization-vaccines-and-biologicals/strategies/global-vaccine-action-plan.
4. Peretti-Watel P, Larson HJ, Ward JK, Schulz WS, Verger P. Vaccine hesitancy: clarifying a theoretical framework for an ambiguous notion. PLoS Curr. 2015; 7. PMID: 25789201
5. Gerussi V, Peghin M, Palese A, Bressan V, Visintini E, Bontempo G, et al. Vaccine Hesitancy among Italian Patients Recovered from COVID-19 Infection towards Influenza and Sars-Cov-2 Vaccination. Vaccines [Basel]. 2021; 9[2]. https://doi.org/10.3390/vaccines9020172 PMID: 33670661
6. Ahmed N, Quinn SC, Hancock GR, Freimuth VS, Jamison A. Social media use and influenza vaccine uptake among White and African American adults. Vaccine. 2018; 36[49]:7556–61. https://doi.org/10.1016/j.vaccine.2018.10.049 PMID: 30389192
7. Galarce EM, Minsky S, Viswanath K. Socioeconomic status, demographics, beliefs and A[H1N1] vaccine uptake in the United States. Vaccine. 2011; 29[32]:5284–9. https://doi.org/10.1016/j.vaccine.2011.05.014 PMID: 21621577
8. Hwang J. Health Information Sources and the Influenza Vaccination: The Mediating Roles of Perceived Vaccine Efficacy and Safety. J Health Commun. 2020; 25[9]:727–35. https://doi.org/10.1080/10810730.2020.1840675 PMID: 33186091
9. Kwok KO, Li KK, Wei WI, Tang A, Wong SYS, Lee SS. Influenza vaccine uptake, COVID-19 vaccination intention and vaccine hesitancy among nurses: A survey. Int J Nurs Stud. 2020; 114:103854. https://doi.org/10.1016/j.ijnurstu.2020.103854 PMID: 33326864
10. Ryan KA, Filipp SL, Gurka MJ, Zirulnik A, Thompson LA. Understanding influenza vaccine perspectives and hesitancy in university students to promote increased vaccine uptake. Heliyon. 2019; 5[10]:e02604. https://doi.org/10.1016/j.heliyon.2019.e02604 PMID: 31667418

11. Suryadevara M, Bonville CA, Rosenbaum PF, Domachowske JB. Influenza vaccine hesitancy in a low-income community in central New York State. Hum Vaccin Immunother. 2014; 10[7]:2098–103. https://doi.org/10.4161/hv.28803 PMID: 25424822

12. Karpman M, Kenney GM, Zuckerman S, Gonzalez D, Courtot B. Confronting COVID-19 vaccine hesitancy among nonelderly adults. Robert Wood Johnson Foundation and Urban Institute. 2021.

13. Khubchandani J, Sharma S, Price JH, Wiblishauser MJ, Sharma M, Webb FJ. COVID-19 Vaccination Hesitancy in the United States: A Rapid National Assessment. J Community Health. 2021; 46[2]:270–7. https://doi.org/10.1007/s10900-020-00958-x PMID: 33389421

14. Olagoke AA, Olagoke OO, Hughes AM. Intention to Vaccinate Against the Novel 2019 Coronavirus Disease: The Role of Health Locus of Control and Religiosity. J Relig Health. 2021; 60[1]:65–80. https://doi.org/10.1007/s10943-020-01090-9 PMID: 33125543

15. Viswanath K, Bekalu M, Dhawan D, Pinnamaneni R, Lang J, McLoud R. Individual and social determinants of COVID-19 vaccine uptake. BMC Public Health. 2021; 21[1]:818. https://doi.org/10.1186/s12889-021-10862-1 PMID: 33910558

16. Roberts HA, Clark DA, Kalina C, Sherman C, Brislin S, Heitzeg MM, et al. To vax or not to vax: Predictors of anti-vax attitudes and COVID-19 vaccine hesitancy prior to widespread vaccine availability. Plos one. 2022; 17[2]:e0264019. https://doi.org/10.1371/journal.pone.0264019 PMID: 35167612

17. Eysenbach G. Improving the Quality of Web Surveys: The Checklist for Reporting Results of Internet E-Surveys [CHERRIES]. J Med Internet Res. 2004; 6[3]:e34. https://doi.org/10.2196/jmir.6.3.e34 PMID: 15471760

18. Belsley DA, Kuh E, Welsch RE. Regression diagnostics: Identifying influential data and sources of collinearity. New York: John Wiley & Sons; 1980. 292 p.

19. Diesel J, Sterrett N, Dasgupta S, Kriss JL, Barry V, Esschert KV, et al. COVID-19 vaccination coverage among adults—United States, December 14, 2020–May 22, 2021. Morbidity and Mortality Weekly Report. 2021; 70[25]:922. https://doi.org/10.15585/mmwr.mm7025e1 PMID: 34166331

20. Lo Moro G, Cugudda E, Bert F, Raco I, Siliquini R. Vaccine Hesitancy and Fear of COVID-19 Among Italian Medical Students: A Cross-Sectional Study. Journal of Community Health. 2022:1–9.

21. Zychlinsky Scharff A, Paulsen M, Schaefer P, Tanisik F, Sugianto RI, Stanislawski N, et al. Students' age and parental level of education influence COVID-19 vaccination hesitancy. European journal of pediatrics. 2021:1–6. https://doi.org/10.1007/s00431-021-04343-1 PMID: 34935085

22. Litaker JR, Tamez N, Lopez Bray C, Durkalski W, Taylor R. Sociodemographic Factors Associated with Vaccine Hesitancy in Central Texas Immediately Prior to COVID-19 Vaccine Availability. International Journal of Environmental Research and Public Health. 2022; 19[1]:368.

23. Khairat S, Zou B, Adler-Milstein J. Factors and Reasons Associated with Low COVID-19 Vaccine Uptake among Highly Hesitant Communities in the US. American journal of infection control. 2022. https://doi.org/10.1016/j.ajic.2021.12.013 PMID: 34995722

24. Okoli GN, Lam OLT, Racovitan F, Reddy VK, Righolt CH, Neilson C, et al. Seasonal influenza vaccination in older people: A systematic review and meta-analysis of the determining factors. PLOS ONE. 2020; 15[6]:e0234702. https://doi.org/10.1371/journal.pone.0234702 PMID: 32555628

25. Schmid P, Rauber D, Betsch C, Lidolt G, Denker ML. Barriers of Influenza Vaccination Intention and Behavior—A Systematic Review of Influenza Vaccine Hesitancy, 2005–2016. PLoS One. 2017; 12[1]:e0170550. https://doi.org/10.1371/journal.pone.0170550 PMID: 28125629

26. Chu A, Gupta V, Unni EJ. Utilizing the Theory of Planned Behavior to determine the intentions to receive the influenza vaccine during COVID-19: A cross-sectional survey of US adults. Preventive Medicine Reports. 2021; 23:101417. https://doi.org/10.1016/j.pmedr.2021.101417 PMID: 34094818

27. Kiviniemi MT, Orom H, Hay JL, Waters EA. Prevention is political: political party affiliation predicts perceived risk and prevention behaviors for COVID-19. BMC public health. 2022; 22[1]:1–9.

28. Fowler EF, Gollust SE. The Content and Effect of Politicized Health Controversies. The ANNALS of the American Academy of Political and Social Science. 2015; 658[1]:155–71.

29. Quinn SC, Jamison AM, Freimuth VS. Measles outbreaks and public attitudes towards vaccine exemptions: some cautions and strategies for addressing vaccine hesitancy. Human Vaccines & Immunotherapeutics. 2020; 16[5]:1050–4. https://doi.org/10.1080/21645515.2019.1646578 PMID: 31403354

30. Bolsen T, Palm R. Politicization and COVID-19 vaccine resistance in the US. Progress in Molecular Biology and Translational Science. 2021.

31. Jennings W, Stoker G, Bunting H, Valgarðsson VO, Gaskell J, Devine D, et al. Lack of trust, conspiracy beliefs, and social media use predict COVID-19 vaccine hesitancy. Vaccines. 2021; 9[6]:593. https://doi.org/10.3390/vaccines9060593 PMID: 34204971

32. Slovic P. Perception of risk. Science. 1987; 236[4799]:280–5. https://doi.org/10.1126/science.3563507 PMID: 3563507

33. Ofei-Dodoo S, Kellerman R, Russell T. Family Physicians' Perception of the New mRNA COVID-19 Vaccines. The Journal of the American Board of Family Medicine. 2021; 34[5]:898–906. https://doi.org/10.3122/jabfm.2021.05.210052 PMID: 34535515

34. Olanipekun T, Abe T, Effoe V, Kagbo-Kue S, Chineke I, Ivonye C, et al. Attitudes and perceptions towards coronavirus disease 2019 [COVID-19] vaccine acceptance among recovered African American patients. Journal of General Internal Medicine. 2021; 36[7]:2186–8. https://doi.org/10.1007/s11606-021-06787-5 PMID: 33904039

35. Violette R, Pullagura GR. Vaccine hesitancy: Moving practice beyond binary vaccination outcomes in community pharmacy. Can Pharm J [Ott]. 2019; 152[6]:391–4. https://doi.org/10.1177/1715163519878745 PMID: 31762851
